# A protein interaction map identifies existing drugs targeting SARS-CoV-2

**DOI:** 10.1186/s40360-020-00444-z

**Published:** 2020-09-03

**Authors:** Claudia Cava, Gloria Bertoli, Isabella Castiglioni

**Affiliations:** 1 Institute of Molecular Bioimaging and Physiology, National Research Council (IBFM-CNR), Via F. Cervi 93, 20090 Segrate-Milan, Milan, Italy; 2 Department of Physics “Giuseppe Occhialini”, University of Milan-Bicocca Piazza dell’Ateneo Nuovo, 1 - 20126 Milan, Italy

**Keywords:** COVID-19, SARS-CoV-2, Drug, Network, In silico analysis, Molecular docking

## Abstract

**Background:**

Severe acute respiratory syndrome coronavirus (SARS-CoV-2), an emerging Betacoronavirus, is the causative agent of COVID-19. Angiotensin converting enzyme 2 (ACE2), being the main cell receptor of SARS-CoV-2, plays a role in the entry of the virus into the cell. Currently, there are neither specific antiviral drugs for the treatment or preventive drugs such as vaccines.

**Methods:**

We proposed a bioinformatics analysis to test in silico existing drugs as a fast way to identify an efficient therapy. We performed a differential expression analysis in order to identify differentially expressed genes in COVID-19 patients correlated with ACE-2 and we explored their direct relations with a network approach integrating also drug-gene interactions. The drugs with a central role in the network were also investigated with a molecular docking analysis.

**Results:**

We found 825 differentially expressed genes correlated with ACE2. The protein-protein interactions among differentially expressed genes identified a network of 474 genes and 1130 interactions.

**Conclusions:**

The integration of drug-gene interactions in the network and molecular docking analysis allows us to obtain several drugs with antiviral activity that, alone or in combination with other treatment options, could be considered as therapeutic approaches against COVID-19.

## Background

Coronaviruses are a group of single-stranded RNA viruses with a wide range of vertebrate hosts.

Coronavirus infection in humans is characterized by a wide spectrum of clinical manifestations ranging from mild influenza symptoms to acute respiratory syndrome with damages in lung function, pneumonia, arrhythmia, or death [1].

Betacoronavirus (β-CoV) is the second of four genera of coronaviruses: alpha, beta, gamma and delta. Over the past two decades, three highly pathogenic human betacoronaviruses have spread from animals to humans: Severe Acute Respiratory Syndrome Coronavirus (SARS-CoV) in 2002–2003, MERS-CoV in 2012 and SARS-CoV-2 in 2019. Compared to SARS-CoV or MERS-CoV, SARS-CoV-2 spreads more rapidly making it difficult to control [1].

Recently, a first model of infection has been proposed [2] in which, after an initial phase of viral exposure, the local innate immune response generates natural IgA and IgM antibodies. In this first stage (5–6 days after infection) patients show their first symptoms limited to upper airways (cough, sore throat) with fever, fatigue and muscle ache. If SARS-CoV-2 (the virus of COVID-19) blocks the innate immunity, it spreads initially from the upper airways to the alveoli, causing pneumonia and dyspnea, and releasing high amounts of viral particles. While many subjects remain asymptomatic or with mild symptoms, in other individuals the response of adaptive immunity, releasing IgM and IgG antibodies, causes severe symptoms such as acute respiratory distress syndrome (ARDS), due to spreading of inflammation, often leading to complications (dyspnea, respiratory failure, fulminant myocarditis) that require the use of intensive therapy. In the last phase, COVID-19 could cause death due to ARDS, acute cardiac and kidney injury, sepsis, and secondary infections. High mortality has been observed in patients with older age or comorbidities (hypertension, diabetes, cardiovascular disease, chronic lung disease and cancer).

Several studies reported that the first sites of infection seem to hit pneumocytes and enterocytes of small intestine [3]. The molecular mechanisms of SARS-CoV-2 entry, as suggested also for SARS-CoV [4], is mediated by the binding of the viral spike glycoprotein to the angiotensin-converting-enzyme-2 (ACE2) receptor, in collaboration with a serine protease, TGRBSS2. This allows the viral capsid fusion with the cell membrane [4].

SARS-CoV-2 is a new virus for our immune system. The IgG antibodies produced by other β-CoV do not recognise this SARS-CoV-2, making this virus currently invincible. A possibility would be the use of immunoglobulin collected from immunized COVID-19 patients to be injected in patients suffering from COVID-19 [5]. In silico approaches on the study of the binding of existing antiviral drugs on cellular receptors to block viral protein entrance could be a fast and low-cost method to test a large amount of possible therapies.

Furthermore, immunotherapy approach combined with antiviral drugs detected by in silico analyses may be a stronger treatment until definitive options such as vaccines are available.

In this work we propose in silico study to identify genes deregulated in COVID-19 positive patients correlated with ACE2. Then, we performed a network analysis based on protein-protein interactions with the aim of identifying existing drugs targeting SARS-CoV-2 deregulated genes, to be proposed as candidate drugs for COVID-19, alone or in combination with other therapeutic strategies. The protein interactions that emerge from our study could help to decipher their mechanisms of action with respect complex biological processes at the basis of COVID-19 infection and progression. The drugs obtained were also investigated through docking simulations.

## Methods

### Gene expression datasets

We collected the gene expression levels of lung tissues infected by SARS-CoV-2 from the Gene Expression Omnibus (GEO) database. Specifically, we selected from GSE147507 2 lung biopsies from postmortem COVID-19 positive male patients and 2 lung biopsies from COVID-19 negative male controls.

Furthermore, from the Genotype-Tissue Expression (GTEx) project we collected gene expression levels of lung tissues from healthy subjects (320 healthy volunteers).

### Differential expression, pathway and network analysis

We normalized and filtered RNA-seq raw counts using the reference of hg19, following the pipeline of the R/ Bioconductor package TCGAbiolinks [6]. In particular, we used the function *TCGAanalyze_Normalization* implementing the EDASeq protocol [7], to perform within-lane and between-lane normalization. We adjusted the data for differences in gene length and distributional differences between samples [7].

Differential expression analysis between lung biopsies from postmortem COVID-19 positive male patients and negative male controls was performed on GSE147507 gene expression levels with the R-package TCGABiolinks [6]. Specifically, we used a generalized linear model which is similar to linear model, but it assumes the negative binomial distribution of RNA-Seq counts. *P*-values, generated from the differential expression analysis, were corrected using the Benjamini–Hochberg procedure for multiple-testing correction [6]. We defined the differentially expressed genes if FDR < 0.01 and |log. FC| > 1.

Pathway enrichment analysis was performed with the R-package clusterProfiler [8]. In particular, we performed an enrichment test for KEGG pathways based on hypergeometric distribution [9]. We identified biological functions that are over-represented in the list of differentially expressed genes [10].

We generated a protein-protein interaction network considering the direct connections among differentially expressed genes in COVID-19 using SpidermiR [11] and we selected the network with the most connected nodes. We calculated for each node the degree centrality (d.c.) that indicates how many neighbours a node has. This index is usually used to identify the nodes that have a central role in the network [11].

Drug-gene interactions were derived by the package rDGIdb [12].

### Correlation

A correlation analysis was performed between ACE2 and the other genes in GTEx data (lung tissues from normal volunteers) to obtain the genes co-expressed with ACE2. The corresponding *p*-values of the Pearson’s correlation were considered and only ACE2 and genes significantly correlated (p-value < 0.001) were used for the subsequent analysis.

### Community detection

We implemented the fast greedy modularity optimization algorithm for finding community structure of our network [13]. Community detection algorithms refer to the procedures for finding groups of connected nodes in a network based on their structural properties [13].

Indeed, a network can be divided into communities or clusters and each community is characterized by many edges within communities and only a few edges between communities.

### Molecular modelling: ligands and protein structures preparation

Docking study explored the binding mode of reported drugs on the 3D model of protease of SARS-CoV-2.

Based on the above analysis, the structural coordinates of potential drugs (file SDF) were downloaded from PubChem [14]. DiscoveryStudio Visualizer was used to generate protein data bank (PDB) format of the ligand from its 3D structure (file SDF) [15] downloaded by PubChem.

Ligand preparation and molecular modelling are performed using AUTODOCK 1.5.6 [15] following the procedure in [16].

After, the ligand is loaded, AUTODOCK automatically prepares it for molecular docking. The software computes Gasteiger charges and if the charges are all zero, it will add charges [16, 17].

Crystal structures of SARS-CoV-2 main proteases (PDB IDs: 5R7Y, 5R7Z, 5R80, 5R81 and 5R82) were obtained from Protein Data Bank. Polar hydrogen atoms were added, and water molecules were removed to the SARS-CoV-2 model.

The macromolecules (main protease) and ligands were saved in pdbqt format to be used for docking analysis. The grid parameters in the x, y and z dimensions were set to 90,90,90. Autodock uses the Lamarckian genetic algorithm to search for the ligand exposure with the highest binding affinity. Default settings were used for other parameters following the docking protocol reported in [16].

Binding energies between ligand and main protease (docking scores) are reported in kcal/mol. High negative values suggest a better binding [16, 17].

Protein-Ligand Interaction Profiler was used to visualize the binding interactions of the selected drugs with 3D model of protease of COVID-19 [18].

## Results

### Differential expression and pathway analysis reveal novel aspects of SARS-CoV-2 biology

**Fig. 1:**
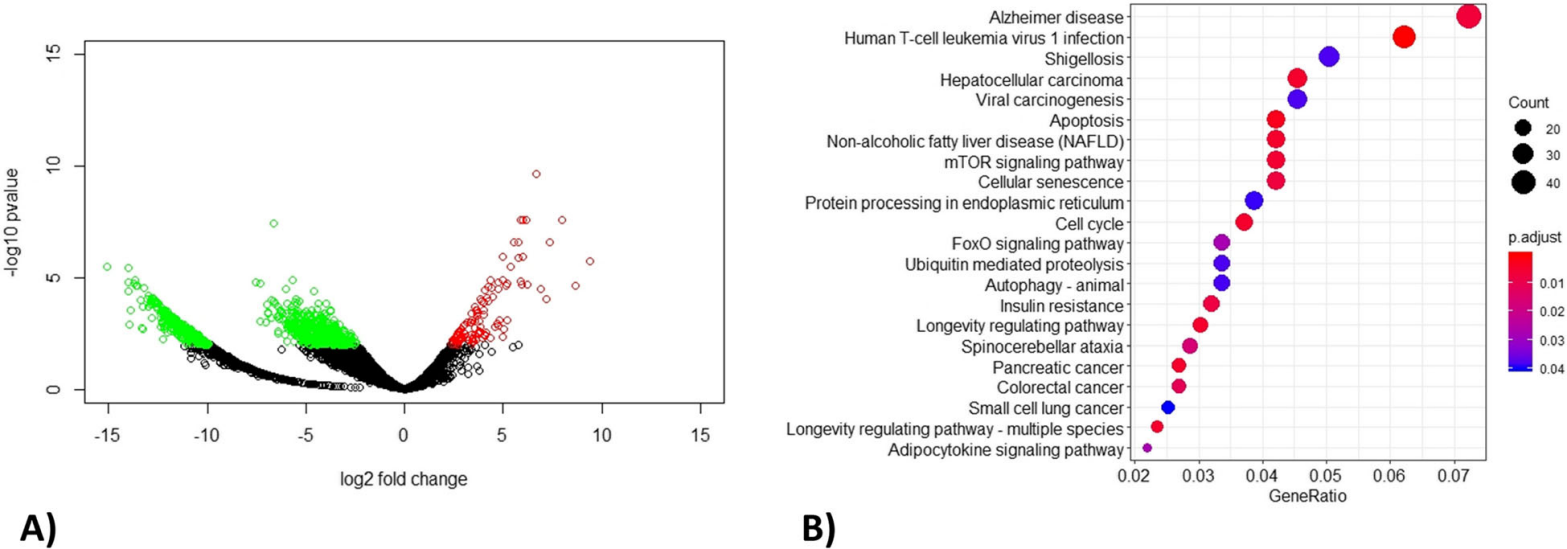
**a** Volcano plot for differential gene expression. The dots represent the genes that are related to *p*-value versus fold change. Red dots are genes that are significantly up-regulated in Covid-19, and green dots are genes significantly down-regulated in Covid-19. **b** Top enriched KEGG pathways. The size of the circles represents the number of differentially expressed gene in the pathway. The color intensity of the circle represents the p-value

Our study revealed 1269 differentially expressed genes between lung biopsies from postmortem COVID-19 positive patients and lung biopsies from negative controls (Supplementary 1). Volcano plot was designed to visualize the distribution of each gene related to the fold change and *p*-value (Fig. 1a).

We found 22 pathways enriched with 1269 differentially expressed genes as reported in Fig. 1b. The top differentially expressed mRNAs were associated with Alzheimer’s disease, human T-cell leukemia virus 1 infection, Shigellosis, hepatocellular carcinoma, viral carcinogenesis, apoptosis, non-alcoholic fatty liver disease (NAFLD), mTOR signaling, cellular senescence, protein processing in endoplasmic reticulum and cell cycle.

### Differentially expressed genes in COVID-19 are correlated with ACE2

From the correlation analysis in GTEx data, we obtained 11,011 genes that correlated with ACE2. We selected differentially expressed genes in COVID-19 that also correlated with ACE2 in GTEX data: 65% of differentially expressed genes (825 genes out of 1269) were found.

### Protein-protein interaction reveals existing drugs targeting SARS-CoV-2

We studied the interactions among differentially expressed genes correlated with ACE2. From the analysis of protein-protein interaction network, we obtained a network of 474 genes and 1130 interactions. We queried DGIdb and we obtained 714 drugs that interact with 119 of 474 genes involving 950 interactions.

**Fig. 2:**
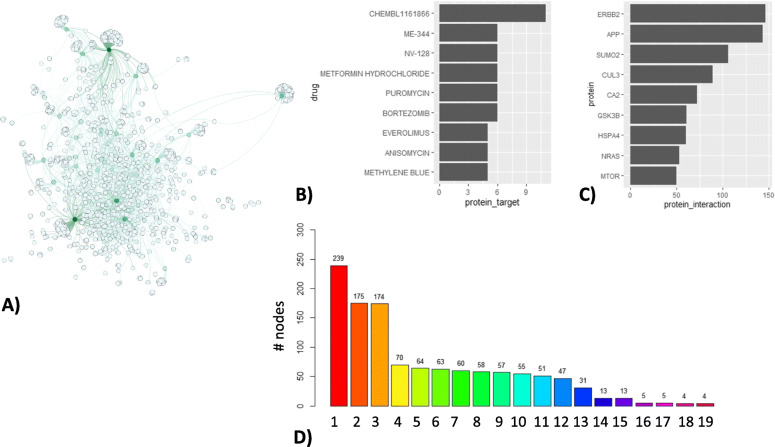
**a** Protein-protein and drug-protein interactions consisting of 474 proteins and 714 drugs. Intensity colour of nodes represents the degree centrality. **b** Drugs with the highest degree centrality in the network. **c** Proteins with the highest degree centrality in the network. **d** Barplot indicating the number of nodes for each community

We integrated protein-protein interactions’ information with the drug-gene interactions obtaining a network of 1188 nodes (including 474 genes and 714 drugs) and 2080 interactions. The overview of such network is shown in Fig. 2a.

The drugs with the highest degree centrality (d.c.) are: CHEMBL1161866 (d.c. = 11), bortezomib (d.c. = 6), puromycin (d.c. = 6), metformin hydrochloride (d.c. = 6), NV-128 (d.c. = 6), ME-344 (d.c. = 6), methylene blue (d.c. = 5), anisomycin (d.c. = 5) and everolimus (d.c. = 5) (Fig. 2b).

The d.c. of the drugs in the network indicates the number of interacting genes. Higher d.c. drugs suggest their central role in the network and the ability to regulate a major number of target genes.

The proteins with the highest d.c. are: ERBB2 (d.c. = 146), APP (d.c. = 143), SUMO2 (d.c. = 106), CUL3 (d.c. = 89), CA2 (d.c. = 72), GSK3B (d.c. = 61), HSPA4 (d.c. = 60), NRAS (d.c. = 53) and MTOR (d.c. = 50) (Fig. 2c).

Moreover, communities detection algorithm identified 19 communities (Fig. 2d), the biggest communities consisting of 239, 175 and 174 nodes (see Fig. 2d).

**Fig. 3:**
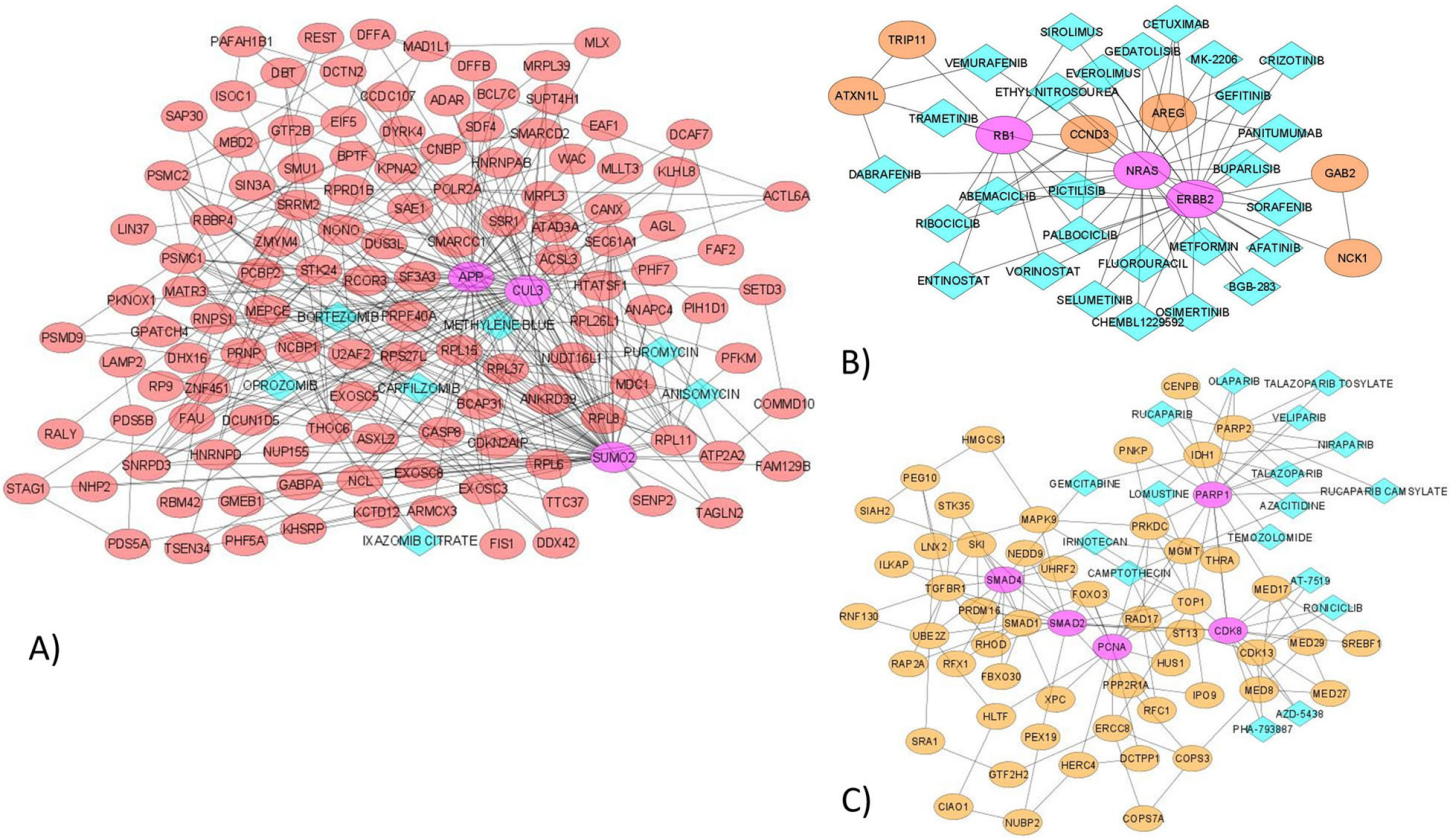
Community detection. The figure shows the biggest communities identified **a** first community, **b** second community, **c** third community. Proteins with higher degree centrality are represented with the purple circles. Drugs are represented with light-blue triangle (we removed the nodes that have only one connection)

In the first community we found 3 genes with the highest d.c.: CUL3 (d.c. = 54), SUMO2 (d.c. = 53), and APP (d.c. = 41). The drugs with key roles in the network are 7: bortezomib, puromycin, ixazomib citrate, carfilzomib, oprozomib, methylene blue and anisomycin. The drugs with the major number of drug targets in the network are: puromycin, methylene blue and anisomycin. They interact with 5 proteins: RPL11, RPL15, RPL26L1, RPL37 and RPL8 (Fig. 3a).

In the second community we found 3 genes with the highest d.c.: ERBB2 (d.c. = 25), NRAS (d.c. = 22) and RB1 (d.c. = 10). The drugs involved in this community are 27: metformin, abemaciclib, ribociclib, palbociclib, pictilisib, fluorouracil, trametinib, vorinostat, sirolimus, entinostat, everolimus, cetuximab, crizotinib, sorafenib, afatinib, ethyl nitrosourea, gefitinib, osimertinib, mk-2206, buparlisib, gedatolisib, bgb-283, panitumumab, selumetinib, chembl1229592, vemurafenib and dabrafenib. The drug with the major number of drug targets in the network is palbociclib, and it interacts with 4 proteins: CCND3, ERBB2, NRAS and RB1 (Fig. 3b).

In the third community we found 5 genes with the highest d.c.: PARP1 (d.c. = 16), PCNA (d.c. = 14) SMAD4 (d.c. = 14), CDK8 (d.c. = 14) and SMAD2 (d.c. = 14). The drugs involved in this community are 17: veliparib, olaparib, rucaparib, niraparib, talazoparib, talazoparib tosylate, rucaparib camsylate, camptothecin, temozolomide, azacitidine, lomustine, gemcitabine, roniciclib, irinotecan, PHA-793887, AT-7519 and AZD-5438. The drug with the major number of drug targets in the network is camptothecin. It interacts with 4 proteins: MAPK9, HUS1, MGMT and TOP1 (Fig. 3c).

### Molecular docking

Docking study was performed on 9 potential drugs (CHEMBL1161866, bortezomib, puromycin, metformin hydrochloride, NV-128, ME-344, methylene blue, anisomycin, and everolimus) against SARS-CoV-2.

The list of drugs examined for docking study is described in Table 1.

**Table 1:**
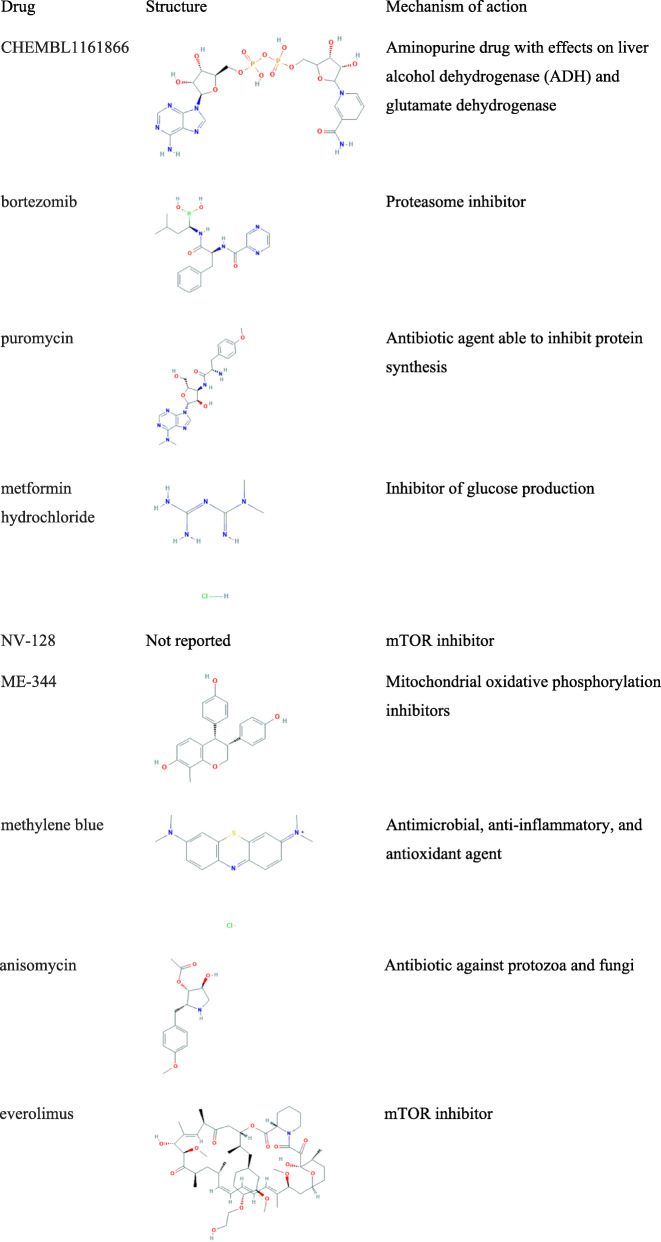
Chemical structure and mechanism of action of the most relevant drugs

The binding energies of CHEMBL1161866 and ME-344 with the main proteases of SAR-CoV-2 are presented in Supplementary 2. CHEMBL1161866 obtained a high binding energy showing a low affinity with the main proteases of SAR-CoV-2. Although ME-344 obtained a good performance, its possible role as drug against COVID-19 is not yet reported in literature.

Two drugs (Puromycin and anisomycin) obtained a negative binding energy value as shown in the Table 2. Puromycin showed promising results in 4 out of 5 protein structures of SARS-CoV-2 with a binding energy lower than − 6.6. Anisomycin was found to interact with 2 out 5 protein structures of SARS-CoV-2 with a binding energy lower than − 6.

**Table 2:** Binding energy of two drugs identified in the docking study

Ligand	Binding Energy kcal/mol				
	5R7Y	5R7Z	5R80	5R81	5R82
puromycin	−6.78	− 6.81	−4.98	− 6.65	−6.94
anisomycin	−5.47	−6.41	−5.44	− 5.6	− 6.08

The details of the parameters for the considered conformations are presented in the Supplementary 2.

**Fig. 4:**
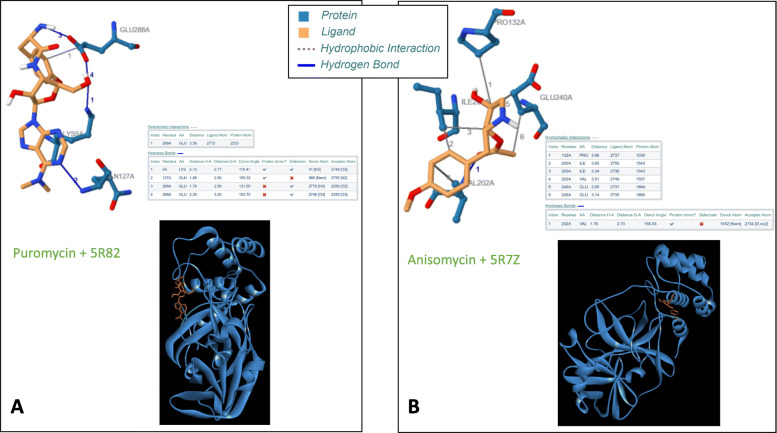
Docking interactions of **a** puromycin with PDB ID 5R82 and **b** anisomycin with PDB ID 5R7Z

Docking interactions of puromycin with PDB ID 5R82 and anisomycin with PDB ID 5R7Z, which obtained the best binding energies, are presented in Fig. 4a-b.

Binding interactions of puromycin with PDB ID 5R82 involve 1 hydrophobic interaction and 4 hydrogen bonds (Fig. 4a). Purine group shows H-bonding interactions with amino acid GLN127A, two hydroxyl groups interact with LYS5A and GLU288A and HN group interacts with GLU288A. Hydrophobic interaction is observed between carbon of methoxyphenyl group and GLU288A.

Binding interactions of anisomycin with PDB ID 5R7Z involve 6 hydrophobic interactions and 1 hydrogen bond (Fig. 4b). VAL202 forms hydrogen bond with ‘O’ of acetate group. Carbon of pyrrolidine interacts by forming hydrophobic interactions with amino acid PRO132A, GLU240A and ILE200A. Carbon of acetate group interacts with GLU240A and carbon of methoxyphenyl interacts with VAL202A and ILE200A.

## Discussion

Our in silico study highlighted protein interactions between genes differentially expressed in COVID-19 patients with respect to negative controls. Such proteins are involved in multiple complexes and biological processes including cell cycle, immune regulation, transcription and protein trafficking. Against these proteins, following in silico tests, we found several drugs, including FDA approved components, as potential treatments for COVID-19.

### Pathway analysis

We found 22 pathways enriched with 1269 differentially expressed genes between lung biopsies from postmortem COVID-19 patients and lung biopsies from negative controls. Intriguing, the top differentially expressed mRNAs were found associated with Alzheimer’s disease.

The risk of contracting COVID-19 in patients with Alzheimer disease (AD) is higher. This is due to their difficulty in following the recommendations from public health authorities and the difficulty of maintaining isolation during behavioural and psychological symptoms of dementia. In addition, patients with AD often have age and comorbid medical conditions associated with a poor prognosis and mortality from COVID-19 [19]. Few publications are currently available on a direct connection between the pathology of AD and COVID-19. From the little we know in the inflammatory response triggered by SARS-CoV-2, during this infection lymphopenia is observed, with loss of CD4 + and CD8 + T cells, hyperproduction of IL6, IL10, IL2R, TNFa and CCL2 [20]. The devastating effect of the cytokine storm on the respiratory system is already known. Less known is the effect of this inflammatory state on the nervous system. Chronic neuroinflammation associated with high levels of cytokine/chemokines has been associated with the pathophysiology of some neurodegenerative diseases (multiple sclerosis, Parkinson’s disease, Alzheimer’s disease, Alzheimer’s disease, Huntington’s disease or amyotrophic lateral sclerosis) [21]. In the case of AD, it has been described that microglia cells lose the ability of degrading Aβ protein in the presence of proinflammatory cytokines (mainly IL1 or IL6), leading to the pathogenic deposits of this protein in the brain [22]. It is thus possible that the cytokine storm produced by SARS-CoV-2 infection could worsen the status of AD patients.

Among the top differentially expressed mRNAs we also found mRNAs associated with human T-cell leukemia virus 1 infection, Shigellosis, hepatocellular carcinoma, viral carcinogenesis, apoptosis, non-alcoholic fatty liver disease (NAFLD), mTOR signaling, cellular senescence, protein processing in endoplasmic reticulum and cell cycle.

Chronic lymphocytic leukemia (CLL) is the most common adult leukemic disorder in the Euro-American population. It is caused by human retrovirus named human T lymphotropic viruses 1 (HTLV-1). HTLV-1 is very similar and relevant to human coronavirus (HCoVs) in animal-to-human transmission event. Although it is not yet fully known how SARS-CoV-2 interacts with host antiviral immunity, similar mechanisms are found from other HCoVs and human pathogenic viruses in other families that are very similar including HTLV-1 and the human immunodeficiency viruses (HIVs) [23]. Several therapeutic options are currently at hand for the first-line or relapsed/refractory CLL, including combination of drugs (i.e., bendamustine, alemtuzumab, ofatumumab) with corticosteroids. Corticosteroids are frequently used to treatment persons with these coronavirus infections in order to counteract high interleukin-6 concentrations, although some studies are still uncertain about the real efficacy and benefit of the use of corticosteroids in COVID-19 patients [24].

Virus infection alters the gut microbiome in humans and makes the organism more susceptible to intestinal bacterial infections such as Shigellosis. Shigellosis is an infectious intestinal disease, also called bacillary dysentery, caused by the genus *Shigella* spp. The expression of ACE2 is abundant in the epithelia of the lungs as well as in intestinal epithelial cells. In line with this scenario, COVID-19 may be associated with gut microbiota [25]. The connection between gastrointestinal tract and the respiratory tract, takes place through shared mucosal immune system which could justify the main symptoms of COVID-19: pneumonia and digestive infections [26, 27].

In addition to the lung and intestine, several evidences showed possible implications of hepatic involvement in SARS-Cov-2 infection. Patients with liver diseases, including hepatocellular carcinoma and Non-alcoholic fatty liver disease (NAFLD) (a metabolic disorder due to the accumulation of fat in the liver), can be more susceptible to the serious consequences of the COVID-19. This could be due to reduced innate immunity to the virus, as the liver plays crucial role in innate and adaptive immunity. As previously reported, the expression of ACE2 is abundant also in the epithelia of intestine, and as there is a rich circulation of blood from the intestine to liver, the spread of the virus in the liver is expected. Reduced hepatic innate immune status could contribute to progression of COVID-19 [28–30].

The mammalian target of rapamycin (mTOR) signaling pathway is a conserved serine/threonine kinase and promotes the cellular proliferation, protein synthesis and DNA replication. mTOR has been already associated with the development of influenza by promoting influenza virus replication [31]. The antiviral properties of mTORs inhibitors have been attributed to a variety of processes. Virus-infected cells initiate the stress response by promoting autophagy to destroy the infecting organism or by activating apoptosis to reduce virus spread. Since mTOR signaling pathway not only inhibits apoptosis but also reduces stress-activated autophagy, mTOR activation could have also a role in vaccine production to enhance virus replication [32, 33].

Apoptosis, cellular senescence, protein processing in endoplasmic reticulum and cell cycle are all processes that are altered as consequence of the virus infection. The infected cell promotes an activation of apoptosis and cell senescence, and an inhibition of protein synthesis and cell cycle in order to remove the infecting organism and reduce the spread of the virus.

### Antiviral proprieties of existing drugs targeting SARS-CoV-2 genes

We investigated the role of existing drugs in the protein network finding the drugs with the highest degree centrality in the network: CHEMBL1161866 (d.c. = 11), bortezomib (d.c. = 6), puromycin (d.c. = 6), metformin hydrochloride (d.c. = 6), NV-128 (d.c. = 6), ME-344 (d.c. = 6), methylene blue (d.c. = 5), anisomycin (d.c. = 5) and everolimus (d.c. = 5) (Fig. 2b).

CHEMBL1161866 is an aminopurine drug with effects on liver alcohol dehydrogenase (ADH) and glutamate dehydrogenase, suggesting a possible beneficial application of this drug on liver infection by SARS-CoV-2. ADH enzymes, highly expressed in liver and colorectal tissues, catalyzed the conversion of alcohol to aldehydes during alcohol metabolism. In particular, ADH could have a role in hepatitis B virus (HBV) and hepatitis C virus infection, being the increased activity of ADH enzymes a clinical marker of liver injury [34]. Although a clear connection between COVID-19 and CHEMBL1161866 has been not proven yet, we could not exclude a possible beneficial effect of this drug on the COVID-19 patients.

Bortezomib (also known as velcade), a proteasome inhibitor approved against multiple myelomas, showed in several studies its antiviral proprieties. Proteasome inhibitors are encouraging antiviral agents that could inhibit viral entry. The ubiquitin-proteasome system (UPS) is involved in different steps of a broad range of viruses’ replication cycle, including coronavirus. The UPS regulates many different processes, such as the cell cycle progression and apoptosis. In addition, ubiquitination is involved in the host immune response to viral infection regulating antigen presentation [35–37]. The Food and Drug Administration (FDA) has approved bortexomib to treat multiple myeloma.

Recent studies suggested that a drug already FDA approved for use against diabetes, metformin, may be effective against COVID-19. Metformin inhibits glucose production decreasing intestinal glucose absorption and enhancing glucose uptake by peripheral tissues. People with diabetes mellitus (DM) have poor prognosis and increased fatality in a wide range of viral infections, including SARS and MERS. The link between COVID-19 and DM could be explained by the use of angiotensin-converting enzyme inhibitors (ACEi) and angiotensin-receptor blockers (ARBs) in people with DM. Indeed, ACEi/ARBs act upregulating the receptor for entry of the virus into host pneumocytes. However, Metformin does not interfere with ACE2, and it is not clear how it could act on patients’ outcome [38, 39]. To date, 20 clinical trials involve metformin as potential treatment of patients with virus diseases. These studies analyzed the effects of metformin in several viral infections such as Chronic Hepatitis C Infection (NCT02972723) and Human Immunodeficiency (NCT04500678).

Methylene blue is a fluorescent dye used for nucleic acid stain. It has been approved by the Food and Drug Administration (FDA) as treatment of methemoglobinemia. Due to its antimicrobial, anti-inflammatory, and antioxidant effects, Methylene blue has been successfully utilized in vitro. Its antiviral proprieties have been attributed to its ability to intercalate into nucleic acids and inactivate RNA viruses. Virus inactivation with Methylene blue was applied in plasma to mitigate the risk of transmission by transfusion in human immunodeficiency virus (HIV), hepatitis B virus, hepatitis C virus and Zika virus [40, 41]. Methylene blue (also known as Provayblue) is indicated by FDA for the treatment of acquired methemoglobinemia. To date, only one on-going clinical trial (NCT04376788) involves Methylene blue as potential treatment of patients with COVID-19. Unfortunately, the results of this clinical study are not yet available.

Anisomycin was originally defined by FDA as an antibiotic against protozoa and fungi. The antiviral effect of anisomycin was demonstrated in cell cultures of Dengue and Zika viruses, mainly involving viral macromolecular synthesis [42]. In addition, anisomycin was reported to inhibit the animal picornavirus encephalomyocarditis virus [43] and to suppress in vitro replication of poliovirus [44] and flavivirus Japanese encephalitis virus [45].

Everolimus is an mTOR inhibitor with well-known antitumor activity in advanced cancer, including kidney and breast cancer. Everolimus showed also in vitro antiviral effects against influenza A virus: in a lethal mouse model of MERS, it delayed death and reduced MERS-CoV infection by ~ 60%. It has also been correlated with a reduced frequency of cytomegalovirus infection in transplant patients [46–48]. FDA approved everolimus for the treatment of neuroendocrine tumors, breast cancer and renal cell carcinoma. To date, 20 clinical trials involve everolimus as potential treatment of patients with viral infections. These studies analyzed the effects of everolimus in several virus diseases such as BK virus (NCT01624948) and hepatitis C virus (NCT01134952).

Puromycin is an antibiotic able to inhibit protein synthesis. Although protein inhibition could be obtained by puromycin treatment, in cornea C-M virus-infected cells as well as in HeLa cells infected with Newcastle disease virus the viral particle continues to be produced due to viral protein intracellular accumulation. In this way, viral particles could be assembled, also in the absence of continuous protein synthesis [49]. It seems that puromycin could be effective only in the initial phases of a viral infection, when the viral protein is necessary to produce the viral particles for the initial virus spreading in the body. Although a clear connection between COVID-19 and puromycin is not reported, we could not exclude a possible beneficial effect of this drug on the COVID-19 patients.

There is not also a clear connection between NV-128, ME-344 and COVID-19, but we cannot exclude a future possible implication of these drugs in COVID-19 patients.

The docking studies estimated that two drugs (puromycin and anisomycin) have potential characteristics of binding to SARS-CoV-2.

## Conclusions

This study identified, in silico, a group of proteins, drug targets of conventional treatments, within multiple complexes and biological processes that could represent effective mechanism of therapeutic actions also for SARS-CoV-2 infection. The protein interactions, that emerge from our analysis, highlight several drugs, as actionable molecules on proteins of the SARS-CoV-2-specific deregulated gene network; they could be proposed with a rational against the virus, alone or in combination with other therapies.

## Supplementary information

**Additional file 1: Supplementary 1.** Differentially expressed genes between lung biopsies from postmortem COVID-19 positive patients and lung biopsies from negative controls. **Supplementary 2.** The details of the parameters for the conformations obtained with molecular docking analysis and the specific binding interactions

## Data Availability

The datasets supporting the conclusions of this article are available in the Gene Expression Omnibus repository (accession number: GSE147507) and Genotype-Tissue Expression (GTEx) project. The links of the datasets are: https://www.ncbi.nlm.nih.gov/geo/query/acc.cgi?acc=GSE147507 (2 lung biopsies from postmortem COVID-19 positive male patients and 2 lung biopsies from COVID-19 negative male controls), https://gtexportal.org/home/(gene expression levels of lung tissues from healthy subjects (320 healthy volunteers)). The crystal structures of SARS-CoV-2 main proteases (accession numbers PDB IDs: 5R7Y, 5R7Z, 5R80, 5R81 and 5R82) were obtained from Protein Data Bank (https://www.rcsb.org/).

## Abbreviations

ARDS: Acute respiratory distress syndrome
ACE2: Angiotensin-converting-enzyme-2
GEO: Gene Expression Omnibus
GTEx: Genotype-Tissue Expression
d.c.: Degree centrality
AD: Alzheimer disease
HCoVs: Human coronavirus
CLL: Chronic lymphocytic leukemia
HTLV-1: Human T lymphotropic viruses 1
mTOR: Mammalian target of rapamycin
DM: Diabetes mellitus
ACEi: Angiotensin-converting enzyme inhibitors
ARBs: Angiotensin-receptor blockers
