# Supplementary material for: A protein interaction map identifies existing drugs targeting SARS-CoV-2
Source: BMC Pharmacol Toxicol. 2020 Sep 3;21:65. doi: 10.1186/s40360-020-00444-z (PMC7470683; doi:10.1186/s40360-020-00444-z)
Supplement: Supplementary file 1 — Additional file 1: Supplementary 1. Differentially expressed genes between lung biopsies from postmortem COVID-19 positive patients and lung biopsies from negative controls. Supplementary 2. The details of the parameters for the conformations obtained with molecular docking analysis and the specific binding interactions [file 40360_2020_444_MOESM1_ESM.pdf]

Conformation 1 Info

```
binding_energy=-6.9
ligand_efficiency=-0.27
inhib_constant=8.7
inhib_constant_units=uM
intermol_energy=-8.4
vdw_hb_desolv_energy=-7.99
electrostatic_energy=-0.41
total_internal=-1.18
torsional_energy=1.49
unbound_energy=-1.18
filename=a.dlg
clRMS=0.0
refRMS=13.8
rseed1=None
rseed2=None
5 hydrogen bonds formed:
5r7y:A:ARG298:HH11 :    ME_344-2: :   0:O2
5r7y:A:GLN110:HE21 :    ME_344-2: :   0:O1
ME_344-2: :   0:H46 :    5r7y:A:ARG105:O
5r7y:A:SER158:HG :    ME_344-2: :   0:O3
ME_344-2: :   0:H44 :    5r7y:A:ASP295:OD1
```

ME-344 5R7Y

Conformation 1 Info

```
binding_energy=-6.7
ligand_efficiency=-0.26
inhib_constant=12.17
inhib_constant_units=uM
intermol_energy=-8.2
vdw_hb_desolv_energy=-7.75
electrostatic_energy=-0.44
total_internal=-1.18
torsional_energy=1.49
unbound_energy=-1.18
filename=a.dlg
clRMS=0.0
refRMS=13.88
rseed1=None
rseed2=None
5 hydrogen bonds formed:
5r7z:A:GLN110:HE21 :    ME_344-2: :   0:O1
5r7z:A:SER158:HG :    ME_344-2: :   0:O3
ME_344-2: :   0:H44 :    5r7z:A:ASP295:OD1
ME_344-2: :   0:H46 :    5r7z:A:ARG105:O
5r7z:A:ARG298:HH11 :    ME_344-2: :   0:O2
```

ME-344 5R7Z

Conformation 1 Info

```
binding_energy=-6.53
ligand_efficiency=-0.25
inhib_constant=16.3
inhib_constant_units=uM
intermol_energy=-8.02
vdw_hb_desolv_energy=-7.92
electrostatic_energy=-0.11
total_internal=-1.12
torsional_energy=1.49
unbound_energy=-1.12
filename=a.dlg
clRMS=0.0
refRMS=12.98
rseed1=None
rseed2=None
3 hydrogen bonds formed:
ME_344-2: :   0:H46 :    5r80:A:ILE152:O
ME_344-2: :   0:H45 :    5r80:A:ASP295:OD1
ME_344-2: :   0:H44 :    5r80:A:ARG105:O
```

ME-344 5R80

Conformation 1 Info

```
binding_energy=-6.83
ligand_efficiency=-0.26
inhib_constant=9.85
inhib_constant_units=uM
intermol_energy=-8.32
vdw_hb_desolv_energy=-7.88
electrostatic_energy=-0.44
total_internal=-1.09
torsional_energy=1.49
unbound_energy=-1.09
filename=a.dlg
clRMS=0.0
refRMS=13.74
rseed1=None
rseed2=None
5 hydrogen bonds formed:
ME_344-2: :   0:H46 :    5r81:A:ARG105:O
ME_344-2: :   0:H44 :    5r81:A:ASP295:OD1
5r81:A:ARG298:HH11 :    ME_344-2: :   0:O2
5r81:A:GLN110:HE21 :    ME_344-2: :   0:O1
5r81:A:SER158:HG :    ME_344-2: :   0:O3
```

ME-344 5R81

Conformation 1 Info

```
binding_energy=-7.05
ligand_efficiency=-0.27
inhib_constant=6.8
inhib_constant_units=uM
intermol_energy=-8.54
vdw_hb_desolv_energy=-8.1
electrostatic_energy=-0.45
total_internal=-1.11
torsional_energy=1.49
unbound_energy=-1.11
filename=a.dlg
clRMS=0.0
refRMS=13.8
rseed1=None
rseed2=None
5 hydrogen bonds formed:
ME_344-2: :   0:H46 :    5r82:A:ARG105:O
ME_344-2: :   0:H44 :    5r82:A:ASP295:OD1
5r82:A:SER158:HG :    ME_344-2: :   0:O3
5r82:A:ARG298:HH12 :    ME_344-2: :   0:O2
5r82:A:GLN110:HE21 :    ME_344-2: :   0:O1
```

ME-344 5R82

Conformation 1 Info

```
binding_energy=-2.38
ligand_efficiency=-0.05
inhib_constant=18.12
inhib_constant_units=mM
intermol_energy=-7.75
vdw_hb_desolv_energy=-7.48
electrostatic_energy=-0.26
total_internal=-7.13
torsional_energy=5.37
unbound_energy=-7.13
filename=a.dlg
clRMS=0.0
refRMS=17.79
rseed1=None
rseed2=None
4 hydrogen bonds formed:
5r7y:A:GLN110:HE21 :  CHEMBL1161866-2: :   0:O15
5r7y:A:ARG105:HN :  CHEMBL1161866-2: :   0:N20
CHEMBL1161866-2: :   0:H58 :    5r7y:A:ARG105:O
5r7y:A:ARG298:HE :  CHEMBL1161866-2: :   0:O16
```

CHEMBL1161866 5R7Y

Conformation 1 Info

```
binding_energy=-4.37
ligand_efficiency=-0.1
inhib_constant=629.08
inhib_constant_units=uM
intermol_energy=-9.74
vdw_hb_desolv_energy=-9.22
electrostatic_energy=-0.52
total_internal=-4.11
torsional_energy=5.37
unbound_energy=-4.11
filename=a.dlg
clRMS=0.0
refRMS=5.5
rseed1=None
rseed2=None
4 hydrogen bonds formed:
5r7z:A:LYS5:HN :  CHEMBL1161866-2: :   0:N21
5r7z:A:GLN127:HN :  CHEMBL1161866-2: :   0:O12
CHEMBL1161866-2: :   0:H59 :    5r7z:A:VAL125:O
CHEMBL1161866-2: :   0:H68 :    5r7z:A:GLN127:O
```

CHEMBL1161866 5R7Z

Conformation 1 Info

```
binding_energy=-2.92
ligand_efficiency=-0.07
inhib_constant=7.23
inhib_constant_units=mM
intermol_energy=-8.29
vdw_hb_desolv_energy=-7.83
electrostatic_energy=-0.46
total_internal=-6.0
torsional_energy=5.37
unbound_energy=-6.0
filename=a.dlg
clRMS=0.0
refRMS=8.24
rseed1=None
rseed2=None
5 hydrogen bonds formed:
CHEMBL1161866-2: :   0:H57 :    5r80:A:GLU288:OE1
5r80:A:GLN127:HN :  CHEMBL1161866-2: :   0:O7
5r80:A:ALA285:HN :  CHEMBL1161866-2: :   0:N21
CHEMBL1161866-2: :   0:H58 :    5r80:A:GLU288:OE2
CHEMBL1161866-2: :   0:H71 :    5r80:A:GLY283:O
```

CHEMBL1161866 5R80

Conformation 1 Info

```
binding_energy=-3.74
ligand_efficiency=-0.09
inhib_constant=1.81
inhib_constant_units=mM
intermol_energy=-9.11
vdw_hb_desolv_energy=-8.42
electrostatic_energy=-0.69
total_internal=-5.46
torsional_energy=5.37
unbound_energy=-5.46
filename=a.dlg
clRMS=0.0
refRMS=7.27
rseed1=None
rseed2=None
4 hydrogen bonds formed:
5r81:A:GLN127:HN :  CHEMBL1161866-2: :   0:O8
5r81:A:LYS5:HZ3 :  CHEMBL1161866-2: :   0:O9,O12
CHEMBL1161866-2: :   0:H72 :    5r81:A:LYS5:O
CHEMBL1161866-2: :   0:H58 :    5r81:A:LEU282:O
```

CHEMBL1161866 5R81

Conformation 1 Info

```
binding_energy=-6.17
ligand_efficiency=-0.14
inhib_constant=29.99
inhib_constant_units=uM
intermol_energy=-11.54
vdw_hb_desolv_energy=-11.03
electrostatic_energy=-0.51
total_internal=-4.35
torsional_energy=5.37
unbound_energy=-4.35
filename=a.dlg
clRMS=0.0
refRMS=8.38
rseed1=None
rseed2=None
3 hydrogen bonds formed:
CHEMBL1161866-2: :   0:H57 :    5r82:A:TYR154:O
5r82:A:GLY11:HN :  CHEMBL1161866-2: :   0:O11
CHEMBL1161866-2: :   0:H72 :    5r82:A:ALA7:O
```

CHEMBL1161866 5R82

Conformation 1 Info

binding_energy=-5.47
ligand_efficiency=-0.29
inhib_constant=98.23
inhib_constant_units=uM
intermol_energy=-7.26
vdw_hb_desolv_energy=-6.05
electrostatic_energy=-1.21
total_internal=-0.66
torsional_energy=1.79
unbound_energy=-0.66
filename=a.dlg
clRMS=0.0
refRMS=22.03
rseed1=None
rseed2=None
2 hydrogen bonds formed:
anisomycin-2: : 0:H25 : 5r7y:A:GLU240:OE1
5r7y:A:VAL202:HN : anisomycin-2: : 0:O3

Anisomycin 5R7Y

Conformation 1 Info

binding_energy=-6.41
ligand_efficiency=-0.34
inhib_constant=20.02
inhib_constant_units=uM
intermol_energy=-8.2
vdw_hb_desolv_energy=-7.45
electrostatic_energy=-0.75
total_internal=-0.72
torsional_energy=1.79
unbound_energy=-0.72
filename=a.dlg
clRMS=0.0
refRMS=18.83
rseed1=None
rseed2=None
1 hydrogen bonds formed:
5r7z:A:VAL202:HN : anisomycin-2: : 0:O3

Anisomycin 5R7Z

Conformation 1 Info

binding_energy=-5.44
ligand_efficiency=-0.29
inhib_constant=103.39
inhib_constant_units=uM
intermol_energy=-7.23
vdw_hb_desolv_energy=-6.65
electrostatic_energy=-0.58
total_internal=-0.79
torsional_energy=1.79
unbound_energy=-0.79
filename=a.dlg
clRMS=0.0
refRMS=20.03
rseed1=None
rseed2=None
2 hydrogen bonds formed:
anisomycin-2: : 0:H25 : 5r80:A:PRO108:O
5r80:A:VAL202:HN : anisomycin-2: : 0:O4

Anisomycin 5R80

Conformation 1 Info

binding_energy=-5.6
ligand_efficiency=-0.29
inhib_constant=78.72
inhib_constant_units=uM
intermol_energy=-7.39
vdw_hb_desolv_energy=-6.66
electrostatic_energy=-0.73
total_internal=-0.56
torsional_energy=1.79
unbound_energy=-0.56
filename=a.dlg
clRMS=0.0
refRMS=19.17
rseed1=None
rseed2=None
2 hydrogen bonds formed:
anisomycin-2: : 0:H25 : 5r81:A:PRO108:O
anisomycin-2: : 0:H28 : 5r81:A:GLU240:OE1

Anisomycin 5R81

Conformation 1 Info

binding_energy=-6.08
ligand_efficiency=-0.32
inhib_constant=34.77
inhib_constant_units=uM
intermol_energy=-7.87
vdw_hb_desolv_energy=-6.47
electrostatic_energy=-1.41
total_internal=-0.4
torsional_energy=1.79
unbound_energy=-0.4
filename=a.dlg
clRMS=0.0
refRMS=22.87
rseed1=None
rseed2=None
3 hydrogen bonds formed:
anisomycin-2: : 0:H28 : 5r82:A:PRO108:O
5r82:A:VAL202:HN : anisomycin-2: : 0:O3
anisomycin-2: : 0:H25 : 5r82:A:GLU240:OE1

Anisomycin 5R82

Conformation 1 Info

binding_energy=-6.78
ligand_efficiency=-0.2
inhib_constant=10.79
inhib_constant_units=uM
intermol_energy=-10.06
vdw_hb_desolv_energy=-7.23
electrostatic_energy=-2.83
total_internal=-3.22
torsional_energy=3.28
unbound_energy=-3.22
filename=a.dlg
clRMS=0.0
refRMS=21.38
rseed1=None
rseed2=None
4 hydrogen bonds formed:
5r7y:A:THR196:HN : Puromycin-2: : 0:O5
Puromycin-2: : 0:H42 : 5r7y:A:GLU240:OE1
Puromycin-2: : 0:H49 : 5r7y:A:GLU240:OE2
Puromycin-2: : 0:H39 : 5r7y:A:GLU240:OE1

Puromycin 5R7Y

Conformation 1 Info

binding_energy=-6.81
ligand_efficiency=-0.2
inhib_constant=10.26
inhib_constant_units=uM
intermol_energy=-10.09
vdw_hb_desolv_energy=-8.17
electrostatic_energy=-1.92
total_internal=-2.58
torsional_energy=3.28
unbound_energy=-2.58
filename=a.dlg
clRMS=0.0
refRMS=11.17
rseed1=None
rseed2=None
3 hydrogen bonds formed:
Puromycin-2: : 0:H42 : 5r7z:A:GLU288:OE1
Puromycin-2: : 0:H39 : 5r7z:A:GLU288:OE1
5r7z:A:LYS137:HZ2 : Puromycin-2: : 0:N8

Puromycin 5R7Z

Conformation 1 Info

binding_energy=-4.98
ligand_efficiency=-0.15
inhib_constant=222.73
inhib_constant_units=uM
intermol_energy=-8.26
vdw_hb_desolv_energy=-6.37
electrostatic_energy=-1.9
total_internal=-3.44
torsional_energy=3.28
unbound_energy=-3.44
filename=a.dlg
clRMS=0.0
refRMS=11.41
rseed1=None
rseed2=None
4 hydrogen bonds formed:
Puromycin-2: : 0:H39 : 5r80:A:LYS137:O
Puromycin-2: : 0:H49 : 5r80:A:GLU290:OE1
Puromycin-2: : 0:H42 : 5r80:A:GLY138:O
Puromycin-2: : 0:H48 : 5r80:A:LYS137:O

Puromycin 5R80

Conformation 1 Info

binding_energy=-6.65
ligand_efficiency=-0.2
inhib_constant=13.37
inhib_constant_units=uM
intermol_energy=-9.93
vdw_hb_desolv_energy=-8.11
electrostatic_energy=-1.82
total_internal=-3.49
torsional_energy=3.28
unbound_energy=-3.49
filename=a.dlg
clRMS=0.0
refRMS=6.18
rseed1=None
rseed2=None
3 hydrogen bonds formed:
5r81:A:LYS5:HZ2 : Puromycin-2: : 0:O4
5r81:A:GLN127:HN : Puromycin-2: : 0:N9,O2
Puromycin-2: : 0:H42 : 5r81:A:GLN127:O

Puromycin 5R81

Conformation 1 Info

binding_energy=-6.94
ligand_efficiency=-0.2
inhib_constant=8.24
inhib_constant_units=uM
intermol_energy=-10.22
vdw_hb_desolv_energy=-8.37
electrostatic_energy=-1.85
total_internal=-3.28
torsional_energy=3.28
unbound_energy=-3.28
filename=a.dlg
clRMS=0.0
refRMS=8.2
rseed1=None
rseed2=None
4 hydrogen bonds formed:
5r82:A:LYS5:HZ3 : Puromycin-2: : 0:O1
Puromycin-2: : 0:H48 : 5r82:A:GLU288:OE2
5r82:A:LYS5:HZ1 : Puromycin-2: : 0:O3
5r82:A:GLN127:HN : Puromycin-2: : 0:N12

Puromycin 5R82

Conformation 1 Info

binding_energy=-5.47
ligand_efficiency=-0.29
inhib_constant=98.23
inhib_constant_units=uM
intermol_energy=-7.26
vdw_hb_desolv_energy=-6.05
electrostatic_energy=-1.21
total_internal=-0.66
torsional_energy=1.79
unbound_energy=-0.66
filename=a.dlg
clRMS=0.0
refRMS=22.03
rseed1=None
rseed2=None
2 hydrogen bonds formed:

Anisomycin 5R7Y

Conformation 1 Info

binding_energy=-6.41
ligand_efficiency=-0.34
inhib_constant=20.02
inhib_constant_units=uM
intermol_energy=-8.2
vdw_hb_desolv_energy=-7.45
electrostatic_energy=-0.75
total_internal=-0.72
torsional_energy=1.79
unbound_energy=-0.72
filename=a.dlg
clRMS=0.0
refRMS=18.83
rseed1=None
rseed2=None
1 hydrogen bonds formed:

Anisomycin 5R7Z

Conformation 1 Info

binding_energy=-5.44
ligand_efficiency=-0.29
inhib_constant=103.39
inhib_constant_units=uM
intermol_energy=-7.23
vdw_hb_desolv_energy=-6.65
electrostatic_energy=-0.58
total_internal=-0.79
torsional_energy=1.79
unbound_energy=-0.79
filename=a.dlg
clRMS=0.0
refRMS=20.03
rseed1=None
rseed2=None
2 hydrogen bonds formed:

Anisomycin 5R80

Conformation 1 Info

binding_energy=-5.6
ligand_efficiency=-0.29
inhib_constant=78.72
inhib_constant_units=uM
intermol_energy=-7.39
vdw_hb_desolv_energy=-6.66
electrostatic_energy=-0.73
total_internal=-0.56
torsional_energy=1.79
unbound_energy=-0.56
filename=a.dlg
clRMS=0.0
refRMS=19.17
rseed1=None
rseed2=None
2 hydrogen bonds formed:

Anisomycin 5R81

Conformation 1 Info

binding_energy=-6.08
ligand_efficiency=-0.32
inhib_constant=34.77
inhib_constant_units=uM
intermol_energy=-7.87
vdw_hb_desolv_energy=-6.47
electrostatic_energy=-1.41
total_internal=-0.4
torsional_energy=1.79
unbound_energy=-0.4
filename=a.dlg
clRMS=0.0
refRMS=22.87
rseed1=None
rseed2=None
3 hydrogen bonds formed:

Anisomycin 5R82

Conformation 1 Info

binding_energy=-6.78
ligand_efficiency=-0.2
inhib_constant=10.79
inhib_constant_units=uM
intermol_energy=-10.06
vdw_hb_desolv_energy=-7.23
electrostatic_energy=-2.83
total_internal=-3.22
torsional_energy=3.28
unbound_energy=-3.22
filename=a.dlg
clRMS=0.0
refRMS=21.38
rseed1=None
rseed2=None
4 hydrogen bonds formed:

Puromycin 5R7Y

Conformation 1 Info

binding_energy=-6.81
ligand_efficiency=-0.2
inhib_constant=10.26
inhib_constant_units=uM
intermol_energy=-10.09
vdw_hb_desolv_energy=-8.17
electrostatic_energy=-1.92
total_internal=-2.58
torsional_energy=3.28
unbound_energy=-2.58
filename=a.dlg
clRMS=0.0
refRMS=11.17
rseed1=None
rseed2=None
3 hydrogen bonds formed:

Puromycin 5R7Z

Conformation 1 Info

binding_energy=-4.98
ligand_efficiency=-0.15
inhib_constant=222.73
inhib_constant_units=uM
intermol_energy=-8.26
vdw_hb_desolv_energy=-6.37
electrostatic_energy=-1.9
total_internal=-3.44
torsional_energy=3.28
unbound_energy=-3.44
filename=a.dlg
clRMS=0.0
refRMS=11.41
rseed1=None
rseed2=None
4 hydrogen bonds formed:

Puromycin 5R80

Conformation 1 Info

binding_energy=-6.65
ligand_efficiency=-0.2
inhib_constant=13.37
inhib_constant_units=uM
intermol_energy=-9.93
vdw_hb_desolv_energy=-8.11
electrostatic_energy=-1.82
total_internal=-3.49
torsional_energy=3.28
unbound_energy=-3.49
filename=a.dlg
clRMS=0.0
refRMS=6.18
rseed1=None
rseed2=None
3 hydrogen bonds formed:

Puromycin 5R81

Conformation 1 Info

binding_energy=-6.94
ligand_efficiency=-0.2
inhib_constant=8.24
inhib_constant_units=uM
intermol_energy=-10.22
vdw_hb_desolv_energy=-8.37
electrostatic_energy=-1.85
total_internal=-3.28
torsional_energy=3.28
unbound_energy=-3.28
filename=a.dlg
clRMS=0.0
refRMS=8.2
rseed1=None
rseed2=None
4 hydrogen bonds formed:

Puromycin 5R82

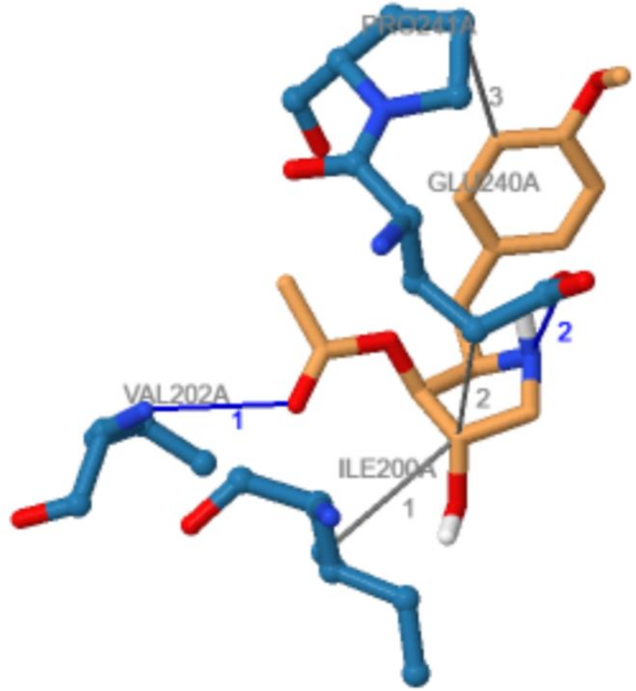

Hydrophobic Interactions

Index	Residue	AA	Distance	Ligand Atom	Protein Atom
1	200A	ILE	3.53	2786	1536
2	240A	GLU	3.45	2786	1887
3	241A	PRO	3.44	2798	1896

Hydrogen Bonds

Index	Residue	AA	Distance H-A	Distance D-A	Donor Angle	Protein donor?	Sidechain	Donor Atom	Acceptor Atom
1	202A	VAL	2.06	3.02	163.36	✔	✖	1545 [Nam]	2783 [O.co2]
2	240A	GLU	2.06	2.80	127.62	✖	✔	2788 [N3]	1889 [O2]

Anisomycin 5R7Y

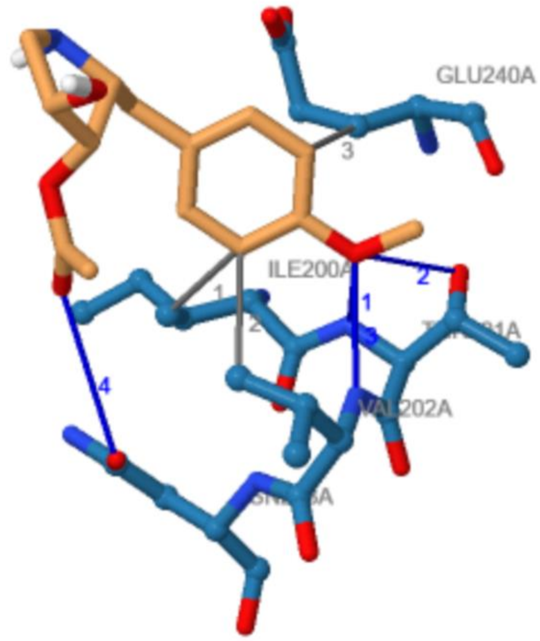

Hydrophobic Interactions

Index	Residue	AA	Distance	Ligand Atom	Protein Atom
1	200A	ILE	2.88	2743	1543
2	202A	VAL	3.17	2743	1557
3	240A	GLU	3.00	2745	1893

Hydrogen Bonds

Index	Residue	AA	Distance H-A	Distance D-A	Donor Angle	Protein donor?	Sidechain	Donor Atom	Acceptor Atom
1	201A	THR	2.75	3.11	102.11	✔	✖	1545 [Nam]	2747 [O3]
2	201A	THR	2.84	3.61	137.63	✖	✔	2747 [O3]	1550 [O3]
3	202A	VAL	2.01	2.94	157.09	✔	✖	1552 [Nam]	2747 [O3]
4	203A	ASN	3.21	3.98	137.03	✖	✔	2730 [O2]	1565 [O2]

Anisomycin 5R80

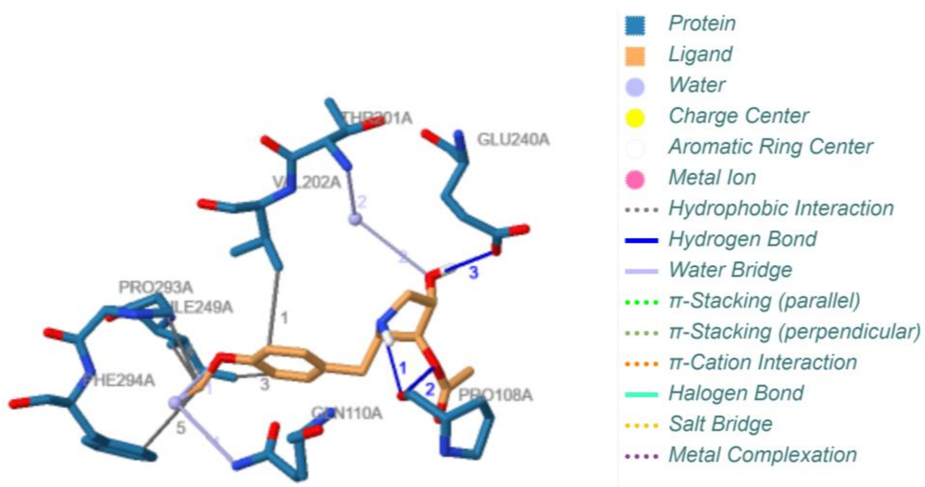

Hydrophobic Interactions

Index	Residue	AA	Distance	Ligand Atom	Protein Atom
1	202A	VAL	3.39	2752	1557
2	249A	ILE	3.59	2757	1968
3	249A	ILE	3.62	2754	1969
4	293A	PRO	3.87	2757	2286
5	294A	PHE	3.40	2757	2295

Hydrogen Bonds

Index	Residue	AA	Distance H-A	Distance D-A	Donor Angle	Protein donor?	Sidechain	Donor Atom	Acceptor Atom
1	108A	PRO	2.09	3.00	147.04	✖	✖	2744 [N3]	853 [O2]
2	108A	PRO	2.35	2.72	101.74	✖	✖	2737 [O3]	853 [O2]
3	240A	GLU	1.90	2.82	156.50	✖	✔	2747 [O3]	1896 [O2]

Water Bridges

Index	Residue	AA	Dist. A-W	Dist. D-W	Donor Angle	Water Angle	Protein donor?	Donor Atom	Acceptor Atom	Water Atom
1	110A	GLN	2.56	3.97	162.34	77.15	✔	869 [Nam]	2756 [O3]	2663
2	201A	THR	3.73	3.17	103.83	80.22	✔	1545 [Nam]	2747 [O3]	2560

Anisomycin 5R81

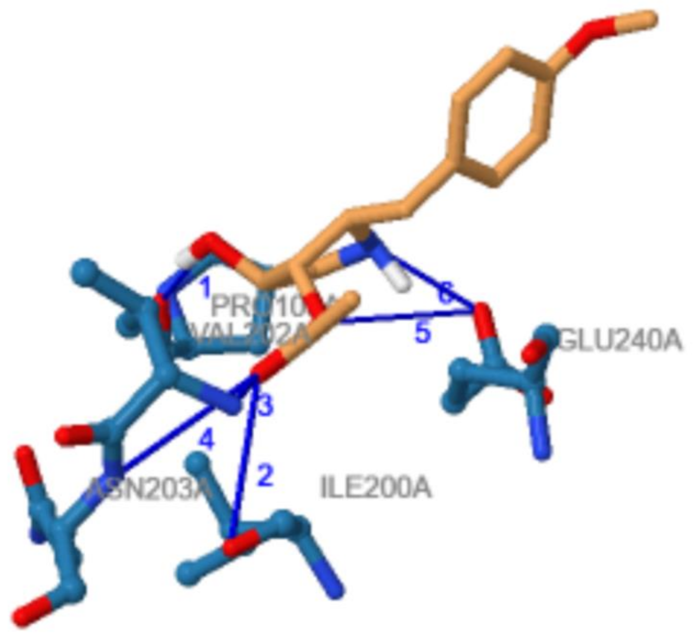

Hydrogen Bonds

Index	Residue	AA	Distance H-A	Distance D-A	Donor Angle	Protein donor?	Sidechain	Donor Atom	Acceptor Atom
1	108A	PRO	1.98	2.90	158.57	✘	✘	2752 [O3]	853 [O2]
2	200A	ILE	3.21	3.63	108.37	✘	✘	2744 [O3]	1540 [O2]
3	202A	VAL	1.82	2.74	154.18	✔	✘	1552 [Nam]	2744 [O3]
4	203A	ASN	3.20	4.06	145.86	✔	✘	1559 [Nam]	2744 [O3]
5	240A	GLU	3.35	4.05	130.71	✘	✔	2742 [O3]	1907 [O2]
6	240A	GLU	1.78	2.65	140.76	✘	✔	2749 [N3]	1907 [O2]

Anisomycin 5R82

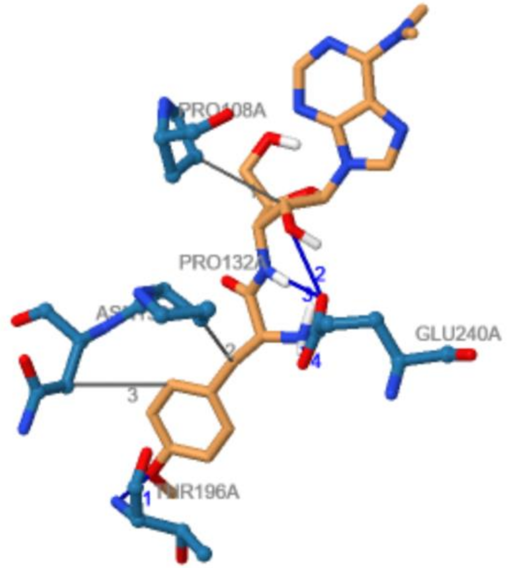

Hydrophobic Interactions

Index	Residue	AA	Distance	Ligand Atom	Protein Atom
1	108A	PRO	3.33	2783	848
2	132A	PRO	3.47	2806	1022
3	133A	ASN	4.00	2812	1029

Hydrogen Bonds

Index	Residue	AA	Distance H-A	Distance D-A	Donor Angle	Protein donor?	Sidechain	Donor Atom	Acceptor Atom
1	196A	THR	2.03	2.98	161.59	✔	✘	1501 [Nam]	2813 [O2]
2	240A	GLU	1.82	2.55	129.17	✘	✔	2818 [O3]	1889 [O2]
3	240A	GLU	1.77	2.78	174.15	✘	✔	2801 [N3]	1889 [O2]
4	240A	GLU	1.67	2.67	165.88	✘	✔	2815 [N3]	1890 [O3]

Puromycin 5R7Y

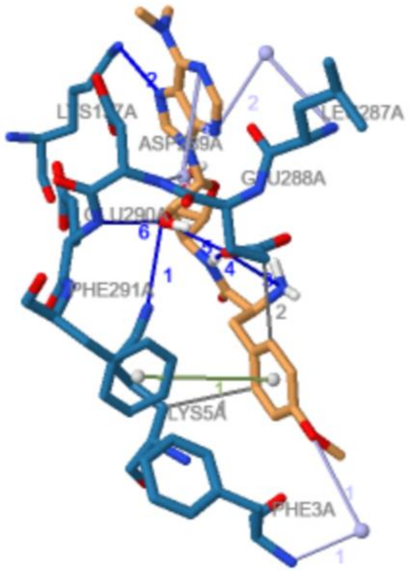

Hydrophobic Interactions

Index	Residue	AA	Distance	Ligand Atom	Protein Atom
1	5A	LYS	3.68	2759	37
2	288A	GLU	3.27	2763	2242

Hydrogen Bonds

Index	Residue	AA	Distance H-A	Distance D-A	Donor Angle	Protein donor?	Sidechain	Donor Atom	Acceptor Atom
1	5A	LYS	2.45	3.22	131.68	✔	✔	41 [N3]	2769 [O3]
2	137A	LYS	1.75	2.74	162.57	✔	✔	1074 [N3]	2742 [N2]
3	288A	GLU	2.71	3.25	113.29	✘	✔	2766 [N3]	2244 [O3]
4	288A	GLU	1.73	2.75	175.83	✘	✔	2752 [N3]	2244 [O3]
5	288A	GLU	1.89	2.82	161.08	✘	✔	2769 [O3]	2244 [O3]
6	290A	GLU	3.39	3.95	117.88	✔	✘	2254 [Nam]	2769 [O3]

Water Bridges

Index	Residue	AA	Dist. A-W	Dist. D-W	Donor Angle	Water Angle	Protein donor?	Donor Atom	Acceptor Atom	Water Atom
1	3A	PHE	3.95	2.88	169.73	88.61	✔	11 [Nam]	2764 [O3]	2568
2	287A	LEU	3.79	3.94	114.34	77.31	✔	2229 [Nam]	2748 [N2]	2608
3	289A	ASP	4.01	3.18	168.20	92.33	✔	2246 [Nam]	2746 [N2]	2626

π-Stacking

Index	Residue	AA	Distance	Angle	Offset	Type	Ligand Atoms
1	291A	PHE	5.44	87.30	1.47	T	2758, 2759, 2760, 2761, 2762, 2763

Puromycin 5R7Z

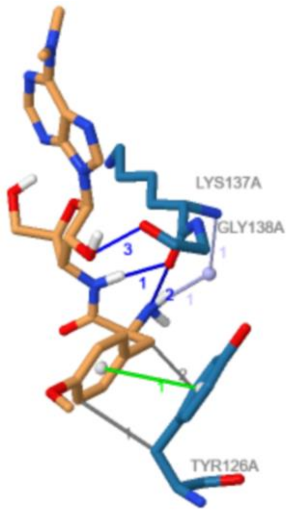

Hydrophobic Interactions ....

Index	Residue	AA	Distance	Ligand Atom	Protein Atom
1	126A	TYR	3.47	2756	978
2	126A	TYR	3.62	2753	980

Hydrogen Bonds —

Index	Residue	AA	Distance H-A	Distance D-A	Donor Angle	Protein donor?	Sidechain	Donor Atom	Acceptor Atom
1	137A	LYS	1.88	2.89	170.72	✖	✖	2748 [Nam]	1069 [O2]
2	137A	LYS	2.08	2.95	142.17	✖	✖	2762 [N3]	1069 [O2]
3	138A	GLY	1.87	2.63	132.09	✖	✖	2765 [O3]	1078 [O2]

Water Bridges —

Index	Residue	AA	Dist. A-W	Dist. D-W	Donor Angle	Water Angle	Protein donor?	Donor Atom	Acceptor Atom	Water Atom
1	137A	LYS	2.86	2.93	169.61	78.28	✔	1066 [Nam]	2762 [N3]	2482

π-Stacking ....

Index	Residue	AA	Distance	Angle	Offset	Type	Ligand Atoms
1	126A	TYR	4.11	25.55	1.77	P	2754, 2755, 2756, 2757, 2758, 2759

Puromycin 5R80

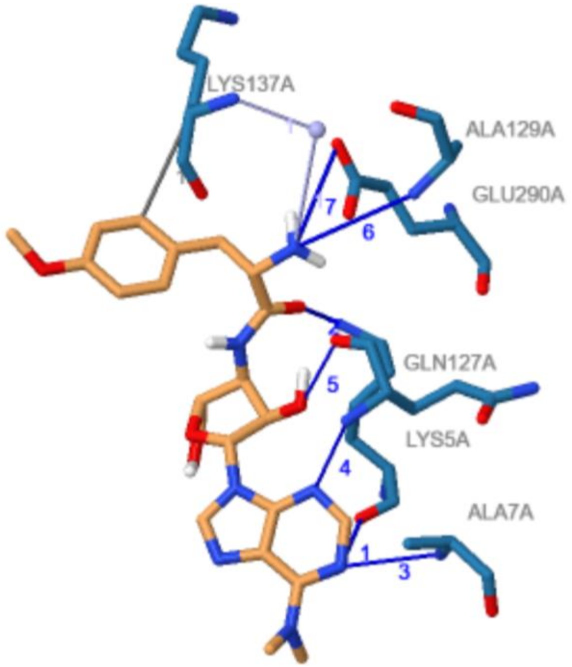

Hydrophobic Interactions ....

Index	Residue	AA	Distance	Ligand Atom	Protein Atom
1	137A	LYS	3.89	2764	1070

Hydrogen Bonds —

Index	Residue	AA	Distance H-A	Distance D-A	Donor Angle	Protein donor?	Sidechain	Donor Atom	Acceptor Atom
1	5A	LYS	2.54	3.38	139.87	✖	✖	2751 [N3]	36 [O2]
2	5A	LYS	2.17	2.95	132.36	✔	✔	41 [N3]	2759 [O3]
3	7A	ALA	2.42	3.04	120.38	✔	✖	50 [Nam]	2751 [N3]
4	127A	GLN	2.10	2.95	144.21	✔	✖	986 [Nam]	2753 [N3]
5	127A	GLN	1.82	2.48	121.83	✖	✖	2774 [O3]	989 [O2]
6	129A	ALA	3.32	4.05	132.40	✔	✖	1001 [Nam]	2771 [N3]
7	290A	GLU	3.07	3.95	146.20	✖	✔	2771 [N3]	2262 [O3]

Water Bridges —

Index	Residue	AA	Dist. A-W	Dist. D-W	Donor Angle	Water Angle	Protein donor?	Donor Atom	Acceptor Atom	Water Atom
1	137A	LYS	3.69	2.99	173.35	77.53	✔	1066 [Nam]	2771 [N3]	2481

Puromycin 5R81
